# Uncoupling the Trade-Off between Somatic Proteostasis and Reproduction in *Caenorhabditis elegans* Models of Polyglutamine Diseases

**DOI:** 10.3389/fnmol.2017.00101

**Published:** 2017-04-20

**Authors:** Netta Shemesh, Nadav Shai, Lana Meshnik, Rotem Katalan, Anat Ben-Zvi

**Affiliations:** Department of Life Sciences, The National Institute for Biotechnology in the Negev, Ben-Gurion University of the NegevBeer Sheva, Israel

**Keywords:** aging, arachidonic acid (AA), *Caenorhabditis elegans*, *lipl-4*, neurodegenerative diseases, proteostasis, polyglutamine (polyQ) diseases, reproduction

## Abstract

*Caenorhabditis elegans* somatic protein homeostasis (proteostasis) is actively remodeled at the onset of reproduction. This proteostatic collapse is regulated cell-nonautonomously by signals from the reproductive system that transmit the commitment to reproduction to somatic cells. Here, we asked whether the link between the reproductive system and somatic proteostasis could be uncoupled by activating downstream effectors in the gonadal longevity cascade. Specifically, we examined whether over-expression of *lipl-4* (*lipl-4(oe)*), a target gene of the gonadal longevity pathway, or increase in arachidonic acid (AA) levels, associated with *lipl-4(oe)*, modulated proteostasis and reproduction. We found that *lipl-4(oe)* rescued somatic proteostasis and postponed the onset of aggregation and toxicity in *C. elegans* models of polyglutamine (polyQ) diseases. However, *lipl-4(oe)* also disrupted fatty acid transport into developing oocytes and reduced reproductive success. In contrast, diet supplementation of AA recapitulated *lipl-4(oe)*-mediated proteostasis enhancement in wild type animals but did not affect the reproductive system. Thus, the gonadal longevity pathway mediates a trade-off between somatic maintenance and reproduction, in part by regulating the expression of genes, such as *lipl-4*, with inverse effects on somatic maintenance and reproduction. We propose that AA could uncouple such germline to soma crosstalk, with beneficial implications protein misfolding diseases.

## Introduction

Aggregates or aggregation intermediates are strongly associated with the etiology of many late-onset neurodegenerative diseases, including Huntington’s disease, amyotrophic lateral sclerosis, Alzheimer’s disease and Parkinson’s disease (Davies et al., 1997; Nussbaum and Polymeropoulos, 1997; Johnston et al., 2000; Glabe and Kayed, 2006; Labbadia and Morimoto, 2015a). In Huntington’s disease, for example, the expansion of polyglutamine (polyQ) repeats is suggested to be the underlying cause of protein misfolding and gain-of-function toxicity (Orr and Zoghbi, 2007). Expression of mutant Huntingtin containing expanded polyQ or even the expanded glutamine tract alone is sufficient to cause cellular dysfunction in various animal models (Zoghbi and Botas, 2002; Sherman and Muchowski, 2003; Voisine and Hart, 2004). Sequestration of misfolded proteins into aggregates is also thought to be part of the cellular defense response against the accumulation of misfolded proteins (Cohen et al., 2006; Tyedmers et al., 2010).

Because chronic expression of misfolded proteins could interfere with and compete for cellular quality control machineries, it was suggested that disruption of protein homeostasis (proteostasis) could be a primary cause for cellular dysfunction and death in neurodegenerative diseases (Gidalevitz et al., 2006; Yerbury et al., 2016). Indeed, expression of aggregation-prone proteins in various model systems was shown to interfere with cellular proteostasis, including disruption of the clearance and folding machineries through competition with other proteins substrates (Suhr et al., 2001; Kim et al., 2002; Venkatraman et al., 2004; Bennett et al., 2007; Bilen and Bonini, 2007; Kitamura et al., 2014). This, in turn, can cause instability of the cellular proteome, affecting the folding of unrelated proteins (Gidalevitz et al., 2006, 2009; Olzscha et al., 2011; Eremenko et al., 2013; Yu et al., 2014; Klabonski et al., 2016). Moreover, changes in chaperone expression levels can disrupt cellular proteostasis and induce an accumulation of damaged protein (Blair et al., 2013; Guisbert et al., 2013; van Oosten-Hawle et al., 2013; Frumkin et al., 2014; Bar-Lavan et al., 2016; Lechler et al., 2017). Thus, maintaining proteostatic capacity is critical for protecting cells from the protein damage associated with protein misfolding diseases.

The challenge to cellular proteostasis is exacerbated in aged individuals as proteostasis maintenance and effective stress response activation decline with age (Taylor and Dillin, 2011; Shai et al., 2014; Labbadia and Morimoto, 2015a). In *Caenorhabditis* *elegans*, proteostatic capacity was shown to *decline* sharply following the onset of reproduction, thereby accelerating the accumulation of polyQ aggregation and toxicity (Ben-Zvi et al., 2009; Liu et al., 2011; Taylor and Dillin, 2013; Labbadia and Morimoto, 2015b; Walther et al., 2015). This decline was, in part, linked to remodeling of the chromatin accessibility of stress gene promoters (Labbadia and Morimoto, 2015b; Merkwirth et al., 2016; Tian et al., 2016). Proteostasis remodeling can be negated by the actions of the gonadal longevity pathway (Lapierre et al., 2011; Vilchez et al., 2012; Shemesh et al., 2013; Shai et al., 2014; Labbadia and Morimoto, 2015b). Signals from the reproductive system can regulate somatic proteostasis in response to inhibition of germline stem cell (GSC) proliferation by activating several transcription factors, including DAF-16/FOXO, SKN-1/Nrf and HSF-1, that are required for proteostasis maintenance during adulthood, as well as for extended lifespan (Hsin and Kenyon, 1999; Libina et al., 2003; Berman and Kenyon, 2006; Antebi, 2012; Shemesh et al., 2013; Steinbaugh et al., 2015; Wang et al., 2017). Thus, the gonadal longevity pathway could determine the investment in somatic maintenance in response to reproduction competence, making the soma available for the demands of reproduction (Kirkwood, 2005; Antebi, 2012; Shai et al., 2014). Given that this trade-off is a regulated switch (Shemesh et al., 2013; Labbadia and Morimoto, 2015b), we asked whether it is possible to uncouple somatic maintenance from reproduction and improve proteostasis without impacting fecundity. We reasoned that over-expression of genes down-regulated by the gonadal longevity pathway would alleviate the protein damage associated with age-dependent neurodegenerative diseases without affecting reproduction.

**Figure 1:**
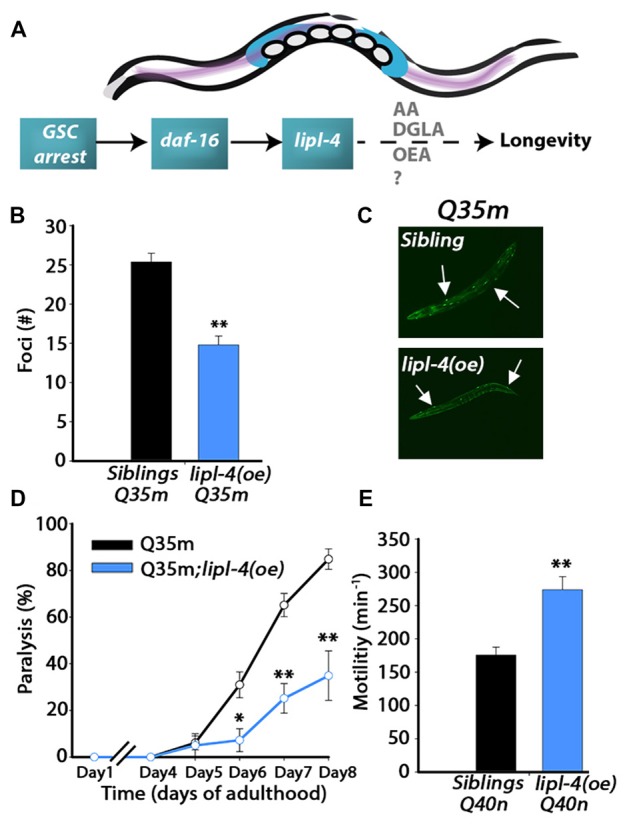
**Over-expression of *lipl-4* postpones the onset of polyglutamine (polyQ) aggregation and toxicity. (A)** Schematic drawing of the gonadal longevity cascade regulating *lipl-4* when germline stem cells (GSCs) are arrested in *C. elegans*. **(B)** The number of bright foci scored on day 2 of adulthood in age-synchronized *Q35m;lipl-4(oe)* animals and their siblings (*n* > 70). **(C)** Representative images of age-synchronized *Q35m;lipl-4(oe)* animals and their siblings on day 2 of adulthood. Arrows indicate foci. **(D)** Motility was scored in age-synchronized *Q35m;lipl-4(oe)* animals and their siblings by determining the percentage of paralyzed animals. **(E)** Motility was scored in age-synchronized *Q40n;lipl-4(oe)* animals and their siblings by counting the number of body bends per minute on day 2 of adulthood. Data was compared to age-matched sibling animals examined under the same condition. *Denotes *P* < 0.05, **denotes *P* < 0.01.

Inhibition of germline proliferation activates DAF-16 that, in turn, induces the expression of a large set of genes. One of the genes up-regulated by DAF-16 is the lysosomal acid lipase-encoding *lipl-4* (Wang et al., 2008; Lapierre et al., 2011; McCormick et al., 2012; Folick et al., 2015; Figure 1A). LIPL-4 itself modulates *C. elegans* lifespan, with its function resulting in the activation of the nuclear hormone receptors NHR-49 and NHR-80 and in the induced expression of autophagy/lipolysis-related genes that modulate the fatty acid metabolism required for *lipl-4*-dependent lifespan extension (Goudeau et al., 2011; Lapierre et al., 2011; Ratnappan et al., 2014; Folick et al., 2015). Moreover, LIPL-4 over-expression results in the enrichment of long-chain fatty acids, including oleoylethanolamide (OEA), ω-6 arachidonic acid (AA) and dihomo-γ-linolenic acid (DGLA) and the ω-3 fatty acid eicosapentaenoic acid (EPA). Diet supplementation of OEA activates NHR-49 and NHR-80, while both DGLA and AA activate autophagy, extending lifespan (O’Rourke et al., 2013; Folick et al., 2015; Figure 1A). Given that *lipl-4* is sufficient and required for lifespan extension (Wang et al., 2008), we asked whether over-expression of LIPL-4 could uncouple proteostasis from reproduction and delay the onset of protein aggregation and toxicity. We found that LIPL-4 modulated the proteostatic switch upon transition to adulthood, resulting in a delay in the onset of aggregation and toxicity in *C. elegans* models of polyQ diseases. However, over-expression of LIPL-4 negatively impacted fatty acid mobilization to the developing oocytes and disrupted reproduction. Surprisingly, diet supplementation of AA improved proteostasis without disrupting reproduction. AA supplementation could, therefore, uncouple somatic maintenance from reproduction, thereby mimicking the beneficial effects of inhibiting germline proliferation on somatic proteostasis without imposing a cost on reproduction.

## Materials and Methods

### Nematodes and Growth Conditions

Nematodes were grown on nematode growth medium (NGM) plates seeded with the *Escherichia coli* OP50-1 strain. Unless otherwise stated, 30–80 embryos, laid at 15°C, were transferred to fresh plates and grown at 25°C for the duration of an experiment. The first day of adulthood (day 1) was set at 50 h after temperature shift, before the onset of egg-laying. Animals were moved every 1–2 days during the reproductive period to avoid progeny contamination. Heat shock-treated animals were discarded after scoring.

### Statistical Analysis

Experiments were repeated at least three times and >15 animals per experimental condition were scored. Data are presented as means ± SEM. *P* values were calculated using the Wilcoxon Mann-Whitney rank sum test to compare two independent populations. *Denotes *P* < 0.05, **denotes *P* < 0.01.

### Foci Quantification

Age-synchronized animals expressing *punc-54::Q35::yellow fluorescent protein (YFP)* (Q35m) were examined using a Leica M165 FC fluorescent stereoscope with a YFP filter and the number of bright foci was counted.

### Paralysis Assay

A total of 15–30 age-synchronized animals were used for each assay. Animals were grown at 25°C for the duration of the experiment. Animals were moved every day, and paralyzed animals were scored by monitoring their movement 5 min after being transferred to a new plate. Animals that did not move were scored as paralyzed (Karady et al., 2013).

### Motility Assay

A total of 15–30 age-synchronized animals were used for each assay. Day 2 adults were moved into M9 buffer and thrashing rates were measured by counting body bends for 15 s. One body bend was defined as a change in the direction of bending at mid-body. Values are presented as bends per minute.

### Stiff Body Paralysis Assay

Animals expressing mutant *unc-52(ts)* were grown at 25°C until day 1 of adulthood, when they were shifted to 15°C and paralysis was scored.

### Thermo-Resistance Assay

Animals were picked at the indicated ages and transferred to a 24-well plate containing heat shock buffer (100 mM Tris-HCl, pH 7.4, 17 mM NaCl and 1% cholesterol supplemented with bacteria). These animals were then subjected to a 37°C heat shock for 6 h. Heat shock buffer was supplemented with SYTOX orange (Invitrogene), and animal survival was scored by monitoring dye uptake, using a Leica M165 FC fluorescent stereoscope with a TXR filter. Fluorescent animals were scored as dead (Karady et al., 2013).

### Heat Shock Treatment

A total of 30–60 age-synchronized animals grown at 25°C were used for each assay. Plates were sealed and placed in a 37°C bath for 90 min. Animals were frozen or fixed immediately following stress.

### RNA Levels

Twenty animals were collected per condition. RNA was extracted using the TRIzol reagent (Invitrogene). For cDNA synthesis, mRNA was reverse-transcribed using the iScript cDNA Synthesis Kit (Bio-Rad). Quantitative PCR was performed on a C1000 Thermal Cycler (Bio-Rad) with KAPA SYBER FAST (KAPA BIOSYSTEMS; Shemesh et al., 2013).

### Progeny Quantification

Individual age-synchronized animals of all tested condition (in parallel) were allowed to lay eggs on fresh plates at 24–25°C. Animals were moved every 24 h during the first 5 days of adulthood (i.e., past the reproduction span) and the number of offspring was scored 48–72 h later. The progeny of >25 animals per genotype were scored.

### Oil-Red-O Staining

Animals were fixed and stained as previously described (O’Rourke et al., 2009) and subsequently mounted and imaged using a Leica DMIL microscope with a 10× 1.0 objective.

### DAPI Staining

Gonad were dissected as in Colaiácovo et al. (2003). Gonads were fixed and stained as previously described (Karady et al., 2013) and subsequently mounted and imaged using an Olympus Fluoview FV1000 confocal microscope through a 60× 1.0 numerical aperture objective with a 405-nm line for excitation.

### Yolk Levels

Age-synchronized animals expressing *pwIs98(YP170::tdimer2)* were fixed as in Karady et al. (2013) and imaged using a Leica M165 FC fluorescent stereoscope with a TXR filter. Pictures were analyzed using imageJ software (NIH).

### Diet Supplementation of Fatty Acids

AA (50 μM dissolved in NP40) and control plates (containing NP40) were prepared as previously described (Deline et al., 2013). A total of 30–80 embryos were transferred to fresh plates and grown at 25°C for the duration of an experiment.

## Results

### *lipl-4* Over-Expression Postponed the Onset of PolyQ-Associated Toxicity

Extended polyQ stretches (>35Q) fused to fluorescent proteins have been used in *C. elegans* as models for polyQ-associated toxicity (Morley et al., 2002; Brignull et al., 2006). To ask whether *lipl-4* over-expression can modulate polyQ aggregation and toxicity, we first crossed animals expressing YFP fused to 35 repeats of glutamine in body wall muscle (Q35m; Morley et al., 2002) with animals expressing *lipl-4* under the regulation of its own promoter as an extra chromosomal array, *lipl-4(oe)*. We then monitored protein aggregation and toxicity and compared the same properties with non-transgenic siblings of *lipl-4(oe)* animals (siblings). By day 2 of adulthood, animals expressing Q35m in the *lipl-4(oe)* background showed 40% less visible foci than did their siblings expressing Q35m alone (Figures 1B,C and Supplementary Figure S1). When we examined motility as a measure of Q35m toxicity, we found that the onset of Q35m-mediated paralysis was delayed in *lipl-4(oe)* animals. By day 8 of adulthood, only 35 ± 11% of *lipl-4(oe)* animals were paralyzed, as compared to 85 ± 4% of their siblings (Figure 1D). Similar results were observed when we crossed animals expressing cyan fluorescent protein fused to 40 repeats of glutamine in neurons (Q40n; Brignull et al., 2006) with animals over-expressing *lipl-4*. The motility of Q40n:*lipl-4(oe)* animals, as measured by thrashing rate (body bends per min), was ~1.6-fold faster than that of their siblings (Figure 1E). Thus, *lipl-4* over-expression modulated the onset and progression of protein aggregation and toxicity in *C. elegans* polyQ disease models.

### *lipl-4* Over-Expression Was Sufficient to Maintain Somatic Proteostasis

**Figure 2:**
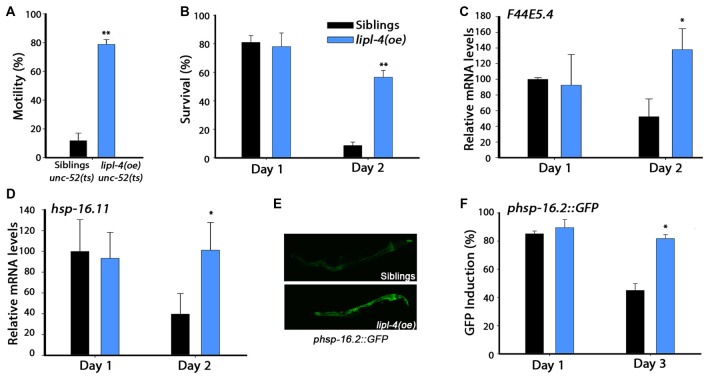
**Over-expression of *lipl-4* maintains proteostasis in adulthood. (A)** Stiff-body paralysis was scored for age-synchronized *lipl-4*(*oe)* animals and their siblings on day 4 of adulthood. **(B)** Thermo-resistance was examined in age-synchronized *lipl-4(oe)* animals and their siblings. Animals were subjected to heat shock (6 h at 37°C) at the indicated times and survival was assayed. **(C,D)** Quantification of *F44E5.4* **(C)** and *hsp-16.11* **(D)** mRNA levels from age-synchronized *lipl-4*(*oe)* animals and their siblings following heat shock (90 min at 37°C). The data presented are normalized to treated siblings on day 1 of adulthood. **(E)** Representative images of age-synchronized *lipl-4(oe)* animals and their siblings expressing *phsp-16.2*::*green fluorescent protein* (*GFP*) that were subjected to heat shock (90 min at 37°C) on day 3 of adulthood. **(F)** Heat shock gene induction was examined in age-synchronized *lipl-4(oe)* animals and their siblings expressing *phsp-16.2::GFP*. Animals were subjected to heat shock (90 min at 37°C) and the percentage of animals expressing GFP was scored. Data was compared to age-matched sibling animals examined under the same condition. *Denotes *P* < 0.05, **denotes *P* < 0.01.

To extend our observations to other aspects of proteostatic function, we next analyzed the impact of *lipl-4(oe)* on the folding of metastable proteins. The ability of *C. elegans* to maintain metastable proteins is dependent on the cellular folding capacity. This ability becomes highly restricted early in adulthood, resulting in age-dependent misfolding (Ben-Zvi et al., 2009). A well-established protein folding reporter is the product of a temperature-sensitive mutation in the gene encoding perlecan, *unc-52(e669, su250; unc-52(ts))*, that causes an age-dependent disruption of muscle organization and motility (Ben-Zvi et al., 2009; Shemesh et al., 2013; Feldman et al., 2014). We, therefore, crossed *unc-52(ts)* mutant animals with animals expressing *lipl-4(oe)* and compared the motility of *unc-52(ts)*;*lipl-4(oe)* with that of their siblings. We found that 79 ± 3% of the *unc-52(ts)*;*lipl-4(oe)* mutant animals were motile on day 4 of adulthood, as opposing to only 12 ± 5% of the *unc-52(ts)* siblings, suggesting that *lipl-4(oe)* can improve proteostasis in adulthood (Figure 2A).

Cellular stress responses, such as the heat shock response, are diminished following the onset of reproduction in *C. elegans*. This is reflected in the sharp decline in the ability of animals to survive various stresses early in adulthood (Ben-Zvi et al., 2009; Shemesh et al., 2013; Labbadia and Morimoto, 2015b). While non-transgenic young adults survived well (81 ± 5%) when challenged by heat shock on day 1 of adulthood, only 9 ± 2.5% survived when first challenged on day 2 of adulthood. In contrast, the survival rates of *lipl-4(oe)* animals remained high, namely 78 ± 9.5% and 56.5 ± 4.7% on days 1 and 2 of adulthood, respectively (Figure 2B). Improved survival rates were also maintained later in life (Supplementary Figure S2A). To determine whether the increased thermo-resistance observed for *lipl-4(oe)* animals is associated with the ability to activate the heat shock response, we compared the stress-dependent induction of heat shock genes, whose expression depends on HSF-1 (*F44E5.4* and *hsp-70*) or on HSF-1 and DAF-16 (*hsp-16.11* and *hsp-16.2*). When animals were challenged by heat shock on day 1 of adulthood, *F44E5.4*, *hsp-70*, *hsp-16.11* and *hsp-16.2* mRNA expression levels were strongly induced in both *lipl-4(oe)* animals and their sibling. In contrast, when animals were heat shocked on day 2 of adulthood, the induced mRNA levels of sibling were 40%–60% lower than in *lipl-4(oe)* animals (Figures 2C,D, Supplementary Figures S2B,C). A transcriptional reporter of *hsp-16.2*, a reporter that regulates green fluorescent protein (GFP) expression in a stress-dependent manner, showed similar behavior. When animals were challenged by heat shock on day 1 of adulthood, strong GFP fluorescence was detected in intestinal cells of both *lipl-4(oe)* animals and their sibling (90 ± 6% and 85 ± 2%, respectively). While the percentage of animals showing high-induction levels of GFP following a heat shock on day 3 of adulthood was maintained for *lipl-4(oe)*, this was not the case for sibling animals (82 ± 3% and 45 ± 5%, respectively; Figures 2E,F). These data suggest that *lipl-4* over-expression is sufficient to modulate stress survival and stress response activation in somatic tissues after the onset of reproduction.

### *lipl-4*-Dependent Rescue of Proteostasis Required the Reproductive System

**Figure 3:**
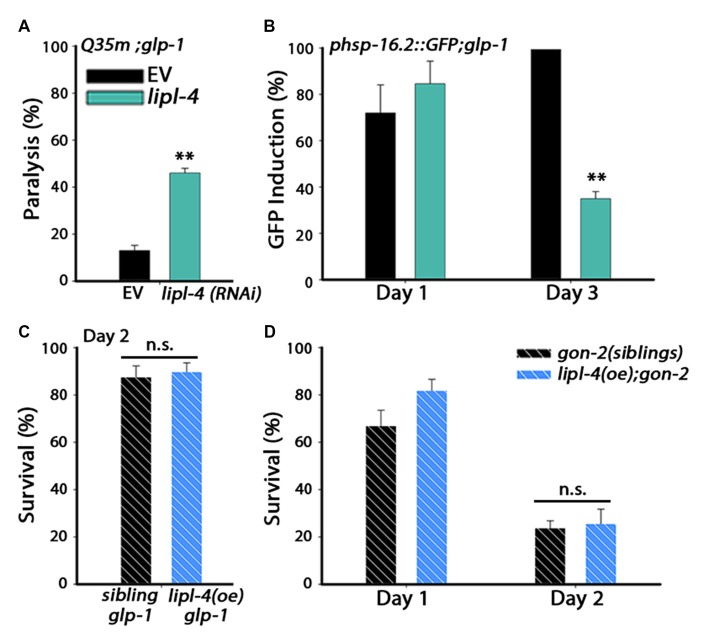
***lipl-4*-dependent rescue of somatic proteostasis requires the somatic gonad.** **(A)** Motility was scored on day 6 of adulthood. Age-synchronized *glp-1* animals expressing Q35m were treated with *lipl-4* or empty vector (EV) control RNAi and the percentage of paralyzed animals was determined. **(B)** Heat shock gene induction was examined. Age-synchronized *glp-1* animals expressing *phsp-16.2*::*GFP* were treated with *lipl-4* or EV control RNAi and subjected to heat shock (90 min at 37°C) on day 1 and day 3 of adulthood. The percentage of animals showing a fluorescent signal in the gut was scored. **(C)** Thermo-resistance was examined in age-synchronized *glp-1;lipl-4(oe)* animals and their siblings. Animals were subjected to heat shock (6 h at 37°C) on day 2 of adulthood and survival was assayed. **(D)** Thermo-resistance was examined in age-synchronized *gon-2*;*lipl-4(oe)* animals and their siblings. Animals were subjected to heat shock (6 h at 37°C) on day 1 and day 2 of adulthoods and survival was assayed. For **(A,B)** data was compared to EV control. For **(C,D)** data was compared to age-matched sibling animals examined under the same condition. (n.s.) no statistical significance, **denotes *P* < 0.01.

In wild type animals, *lipl-4* mRNA levels decline upon transition to adulthood (Supplementary Figure S3A). *lipl-4* is regulated by DAF-16 as part of the gonadal longevity pathway, one of several transcription factors that are activated upon inhibition of GSCs proliferation (Hsin and Kenyon, 1999; Wang et al., 2008; Antebi, 2012; McCormick et al., 2012). Accordingly, *lipl-4* mRNA levels were strongly induced (7-fold) in *glp-1(e2141)* (*glp-1*) germline proliferation mutant animals (Supplementary Figure S3B; Wang et al., 2008; McCormick et al., 2012). We, therefore, asked if down-regulation of *lipl-4* is sufficient to impact germline-dependent rescue of proteostasis. For this, we examined whether *lipl-4(RNAi)* can affect Q35m-associated toxicity and heat shock activation of *glp-1* mutant animals during adulthood. *Q35m;glp-1* animals show reduced aggregation and reduced paralysis during adulthood (Shemesh et al., 2013). However, *Q35m;glp-1* paralysis was induced 3.5-fold when treated with *lipl-4(RNAi)*, as compared to *Q35m;glp-1* animals treated with an empty vector (EV) control (Figure 3A). Likewise, *glp-1* animals that were treated with *lipl-4*(RNAi) lost their ability to induce an effective heat shock response. Induction of *hsp-16.2*-dependent GFP on day 3 of adulthood was reduced 3-fold in *lipl-4*(RNAi)-treated *glp-1* animals, as compared to *glp-1* animals treated with an EV control (Figure 3B). We next ask whether over-expression of *lipl-4* could further enhance proteostasis in *glp-1* animals. *glp-1* mutant animals were crossed with *lipl-4(oe)* animals and subjected to heat shock. Heat shock survival rates of *glp-1;lipl-4(oe)* animals and their siblings were unaffected by *lipl-4(oe)* (87.5 ± 4.8% vs. 89.7 ± 4%, Figure 3C). Thus, LIPL-4 is required for proteostatic remodeling downstream of the longevity reproductive pathway.

LIPL-4 is expressed in the intestine, where it regulates DAF-16 function (Wang et al., 2008; Lapierre et al., 2011; Antebi, 2012; Folick et al., 2015). To determine whether the effects of *lipl-4* on somatic proteostasis require the reproductive system, gonadogenesis-defective *gon-2(q388)*, (*gon-2*) mutant animals were crossed with *lipl-4(oe)*-expressing animals and their ability to survive stress in adulthood was examined. Although *lipl-4(oe)* animals maintained high heat shock survival rates (Figure 2B), *lipl-4(oe);gon-2* animals lost the ability to mount an effective heat shock response by day 2 of adulthood and their survival rates sharply declined from 82 ± 7% to 25.5 ± 6% (Figure 3D), similar to what was seen with the wild type (Figure 2B) and non-transgenic *gon-2* animals (70 ± 7% to 24 ± 7%, Figure 3D). These data indicate that *lipl-4*-dependent rescue of somatic proteostasis required the gonad.

### Over-Expression of *lipl-4* Disrupted Reproduction

**Figure 4:**
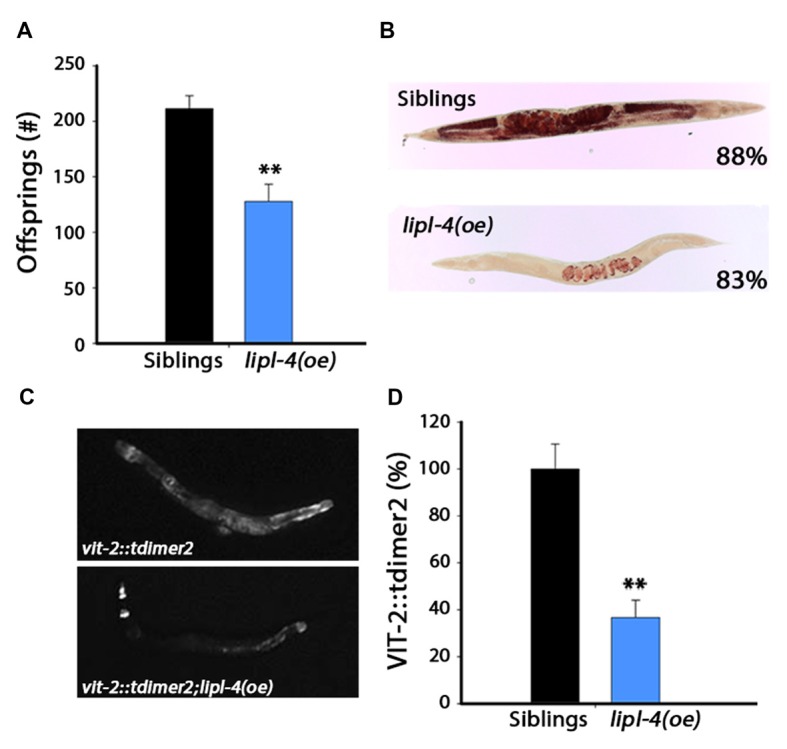
**Over-expression of *lipl-4* disrupts reproduction. (A)** Progeny numbers were scored in age-synchronized *lipl-4(oe)* animals and their siblings. **(B)** Representative images of total fat stores. Age-synchronized *lipl-4(oe)* animals and their siblings were collected on day 2 of adulthood and total fat stores were analyzed using Oil-Red-O staining. Values indicate the percentage of animals showing the presented staining pattern (*n* > 18). **(C)** Representative images of age-synchronized *lipl-4(oe)* animals and their siblings carrying *vit-2::tdimer2* on day 2 of adulthood. **(D)** VIT-2::tdimer2 levels were quantified from images using the ImageJ software and normalized to siblings levels. Data was compared to age-matched sibling animals examined under the same condition. **Denotes *P* < 0.01.

We established that *lipl-4(oe)* could modulate proteostasis and alleviate the protein damage associated with age-dependent misfolding, and that such rescue required the gonad. We next asked whether there is *lipl-4(oe)*-dependent impact on reproduction. *lipl-4(oe)* animals were smaller than their siblings but were not developmentally delayed. Therefore, we first examined the effect of *lipl-4(oe)* on fecundity by determining the number of progeny *lipl-4(oe)* and their siblings produced. Notably, *lipl-4(oe)* had  ~40% less offspring than did their siblings (127 ± 16 and 211 ± 12 offspring, respectively; *p* < 0.0001; Figure 4A). This strong reduction in progeny number was not due to arrest of germline proliferation, since *lipl-4(oe)* and their siblings contained proliferating germ cells (Supplementary Figure S4).

The gonadal longevity cascade modulates fatty acids metabolism and affects the levels of various fatty acids and their distribution in the nematode body (Wang et al., 2008; Goudeau et al., 2011; Lapierre et al., 2011; McCormick et al., 2012; Lapierre et al., 2013; Ratnappan et al., 2014; Steinbaugh et al., 2015). For example, when germline proliferation is inhibited, fatty acids accumulate in the body cavity, where they activate the transcription of genes required for fatty acid mobilization (Lynn et al., 2015; Steinbaugh et al., 2015). To ask how *lipl-4(oe)* could negatively impact reproduction, we first monitored total fat stores in *lipl-4(oe)* animals and their siblings using Oil-Red-O staining. Most of the *lipl-4(oe)* animals (83%) showed a strong reduction in lipid levels, with lipids being mostly located in the developing oocytes (Figure 4B and Supplementary Figure S5).

Levels of vitellogenic proteins are also affected by reproductive signals (DePina et al., 2011). Moreover, over-expression of vitellogenic proteins was previously shown to reduce *lipl-4* levels (Seah et al., 2016). To further elucidate the effect of LIPL-4 on reproduction, we next examined transport of lipids from the intestine to the germline by monitoring the levels and localization of the vitellogenic protein VIT-2 tagged with tdimer2 (VIT-2::tdimer2). Coinciding with diminished fat stores, VIT-2 levels were reduced by ~60% in *lipl-4(oe)* animals, as compared to their siblings, on the day 2 of adulthood (Figures 4C,D). Fatty acid uptake by oocytes was, therefore, disrupted in *lipl-4(oe)* animals, which could, in turn, impact progeny production. Thus, while enhanced *lipl-4* levels improved somatic proteostasis, they were detrimental to reproduction.

### AA Uncouples Somatic Proteostasis from the Reproductive System

**Figure 5:**
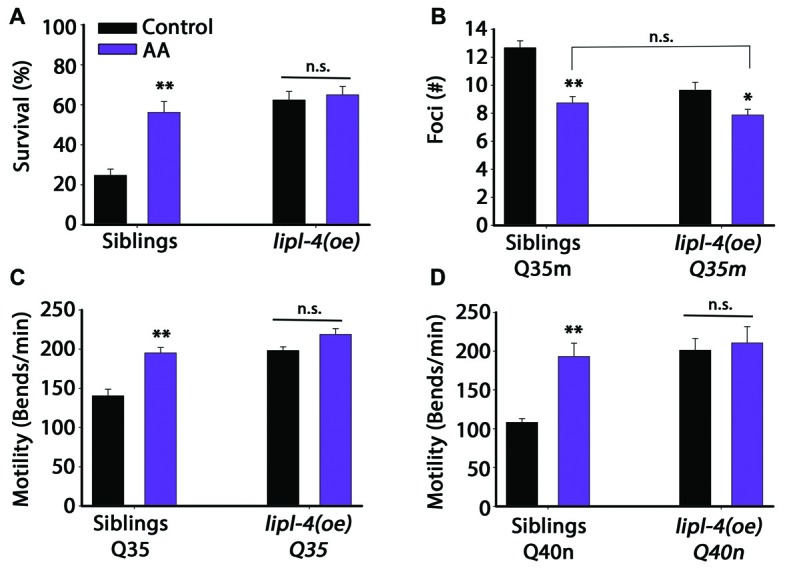
**Diet supplementation of arachidonic acid (AA) could mimic the effects of *lipl-4(oe)* on proteostasis.** **(A)** Thermo-resistance was examined in age-synchronized *lipl-4(oe)* animals and their siblings. Animals were grown on control (NP40) or AA-supplemented nematode growth medium (NGM) plates, subjected to heat shock (6 h at 37°C) and survival was assayed on day 2 of adulthood. **(B)** The number of bright foci was scored in age-synchronized *Q35m;lipl-4(oe)* animals and their siblings (*n* > 40). Animals were grown on control (NP40) or AA-supplemented NGM plates and the number of foci was counted on day 2 of adulthood. **(C,D)** Motility was scored in age-synchronized *Q35m;lipl-4(oe)*, *Q40n;lipl-4(oe)* animals and their siblings. Animals were grown on control (NP40) or AA-supplemented NGM plates and the number of body bends per minute was scored on day 2 of adulthood. Data was compared to control plates (NP40). (n.s.) no statistical significance, *denotes *P* < 0.05, **denotes *P* < 0.01.

Over-expression of *lipl-4* did not uncouple the trade-off between somatic maintenance and reproduction. Metabolite and lipid profiling of *lipl-4(oe)* identified accumulation of several long-chain fatty acids, including OEA, EPA, DGLA and AA (O’Rourke et al., 2013; Folick et al., 2015). While DGLA causes GSC death and complete sterility, EPA does not affect fertility (Watts and Browse, 2006). We, therefore, asked whether modulating *lipl-4(oe)* downstream products could potentially improve proteostasis without disrupting reproduction. We first examined whether diet supplementation of OEA, EPA or AA could regulate heat shock survival, as a simple readout of proteostasis. Embryos were transferred to fatty acid-supplemented or control plates (containing NP40) and their survival was examined on day 2 of adulthood. Diet supplementation of OEA or EPA had no significant effect on animal survival following heat shock on day 2 of adulthood (Supplementary Figure S6A). In contrast, when non-transgenic siblings were supplemented with AA, heat shock survival rates were significantly improved on day 2 of adulthood, as compared to siblings grown on control plates (56 + 5% and 25 + 3%, respectively; Figure 5A). Diet supplementation of AA to *lipl-4(oe)* animals did not further improve survival, as compared to *lipl-4(oe)* animals grown on control plates (65 + 4% and 63 ± 4%, respectively). Likewise, the percentage of sibling animals showing induction of *hsp-16.2*-dependent GFP upon heat shock was increased in animals supplemented with AA, as compared to controls. However, diet supplementation of AA to *lipl-4(oe)* animals did not further increase *hsp-16.2*-dependent GFP expression following heat shock (Supplementary Figure S6B). This was not due to AA-dependent increases in *lipl-4* levels, as diet supplementation of AA did not affect *lipl-4* mRNA levels (Supplementary Figure S7). These data thus support a role for AA in proteostasis remodeling.

We then tested whether diet supplementation of AA could affect Q35m aggregation. A ~30% reduction in foci number was observed in Q35m siblings supplemented with AA. Whereas only a slight reduction in foci formation was observed in *Q35m;lipl-4(oe)* animals supplemented with AA and this reduction was not significantly different from Q35m siblings supplemented with AA (Figure 5B). Similar behavior was observed for Q35m- and Q40n-associated toxicity. *Q35m;lipl-4(oe), Q40n;lipl-4(oe)* animals and their siblings were grown on control or AA-supplemented plates and motility was monitored. Diet supplementation of AA to siblings expressing Q35m or Q40n improved their thrashing rates by 1.4- and 1.8-fold, respectively, compared to animals grown on control plates. In contrast, diet supplementation of AA did not further improve the motility of *Q35m;lipl-4(oe)* or *Q40n;lipl-4(oe)* animals (Figures 5C,D). Thus, diet supplementation with AA mimics the effects of *lipl-4(oe)* on proteostasis, suggesting that the increased AA level, observed in *lipl-4(oe)* animals is sufficient to remodel proteostasis in adulthood, likely via agents downstream of LIPL-4.

**Figure 6:**
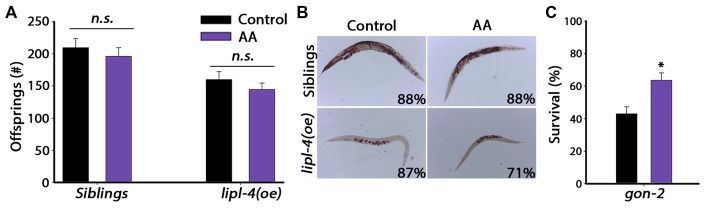
**Reproduction is unaffected by diet supplementation of AA. (A)** Progeny numbers were scored in age-synchronized *lipl-4(oe)* animals and their siblings grown on control (NP40) or AA-supplemented NGM plates. **(B)** Representative images of total fat stores. Age-synchronized *lipl-4(oe)* animals and their siblings were grown on control (NP40) or AA-supplemented NGM plates and collected on day 2 of adulthood. Total fat stores were analyzed using Oil-Red-O staining. Values indicate the percentage of animals showing the presented staining pattern (*n* > 14). **(C)** Thermo-resistance was examined in age-synchronized *gon-2* animals. Animals grown on control (NP40) or AA-supplemented NGM plates were subjected to heat shock (6 h at 37°C) on day 2 of adulthood and survival was assayed. Data was compared to control plates (NP40). (n.s.) no statistical significance, *denotes *P* < 0.05.

Given that AA mimics the effects of *lipl-4(oe)* on somatic proteostasis, we next asked whether AA supplementation has a negative impact on the reproductive system, similar to *lipl-4(oe)*. For this, we examined the effect of AA on progeny production by *lipl-4(oe)* animals and their siblings. A previous study showed that only accumulation of high levels (>20% of their lipids) of AA affected fertility (Watts and Browse, 2006). We, therefore, asked whether diet supplementation of AA at a concentration that improved proteostasis (0.05 mM AA) affected reproduction. AA diet supplementation had no significant effect on the number of progeny produced by *lipl-4(oe)* siblings. Specifically, animals grown on control plates produced 210 ± 14 offspring, while AA-supplemented animals produced 196 ± 13 offspring. Likewise, *lipl-4(oe)* animals grown on control or AA produced similar numbers of offspring (160 ± 12 and 145 ± 10, respectively; Figure 6A). Moreover, diet supplementation of AA to *lipl-4(oe)* animals or their siblings did not affect fatty acid levels or localization, as monitored by Oil-Red-O staining or VIT-2 levels. Most siblings grown on either AA-supplemented or control plates (88%) showed normal fatty acid stores, while *lipl-4(oe)* animals showed low fatty acid levels localized in oocytes, regardless of diet (Figures 4B, 6B). Moreover, AA supplementation to wild type animals did not affect VIT-2 levels (Supplementary Figure S8). These data indicate that diet supplementation of AA can uncouple somatic proteostasis from the reproductive system.

Any benefits derived from AA supplementation in the soma came with no apparent cost to reproduction. We, therefore, asked whether AA-dependent rescue of proteostasis required the reproductive system. To test this directly, we examined the effect of AA supplementation on gonad-less animals by monitoring heat shock survival of *gon-2* animals grown on control or AA-supplemented plates. While the survival of *gon-2* animals grown on control plates was low on day 2 of adulthood (43 ± 4%; Shemesh et al., 2013), survival rates of *gon-2* animals grown on AA-supplemented plates were surprisingly high (63.7 ± 4.4%, Figure 6C), similar to AA-supplemented siblings (Figure 5A). Thus, as opposed to *lipl-4*, the impact of AA on somatic proteostasis does not require the reproductive system. Rather, a small increase in AA levels was sufficient to improved proteostasis in adulthood with no apparent cost to reproduction.

## Discussion

**Figure 7:**
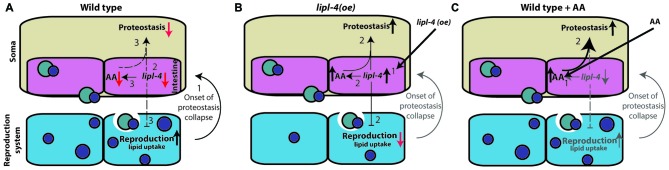
**Signals from the reproductive system regulate somatic proteostasis. (A)** The onset of reproduction regulates proteostasis cell-nonautonomously (1). This activates the gonadal longevity pathway that down regulates *lipl-4* expression at the onset to reproduction (2). When embryo production begins proteostasis sharply declines. This is associated with decreased *lipl-4* and AA levels (3). **(B)** Over-expression of *lipl-4* (1) restores somatic proteostasis and enhances AA levels but also disrupts fatty acid mobilization to developing oocytes and thus disrupt reproduction (2). **(C)** Diet supplementation of AA (1) restores somatic proteostasis (2), like *lipl-4* over-expression, but did not disrupt fatty acid mobilization or reproduction.

Complex signals control the proteostatic switch employed at the onset of reproduction to connect somatic maintenance to the reproductive system (Antebi, 2012). In wild type animals, the onset of egg laying is linked to a sharp decline in the ability to activate stress responses and maintain proteostasis mediated by signals from the gonad (Ben-Zvi et al., 2009; Liu et al., 2011; Taylor and Dillin, 2013; Labbadia and Morimoto, 2015b; Walther et al., 2015). The inhibition of germline proliferation, however, activates the gonadal longevity pathway, resulting in enhanced somatic proteostasis (Shemesh et al., 2013). Here, we showed that this proteostatic switch can be activated upon over-expression of LIPL-4 or AA enrichment, both of which act downstream of the gonadal longevity cascade (Wang et al., 2008; Lapierre et al., 2011; O’Rourke et al., 2013). We propose that the observed decline in *lipl-4* levels upon transition to adulthood is associated with reduced proteostatic capacity but with improved progeny production (Figure 7A). Over-expression of *lipl-4* thus restores somatic proteostasis, yet disrupts fatty acid accumulation in growing oocytes and thus, progeny production (Figure 7B). Because AA diet supplementation resulted in improved somatic proteostasis but did not affect or require the reproductive system, we suggest that the adverse effects of LIPL-4 function on reproduction can be bypassed and that its beneficial effects on somatic proteostasis can be retained by increased AA levels (Figure 7C). The somatic proteostasis rescue mediated by AA could thus delay the onset of toxicity and aggregation in *C. elegans* models of polyQ diseases without cost to reproduction.

### LIPL-4 has Inverse Effects on Reproduction and Somatic Maintenance

The antagonistic pleiotropy theory suggests that genes that have beneficial effects early in life could be selected for, even if they have detrimental effects later in life (Williams, 1957). Given how LIPL-4 has inverse effects on progeny production and proteostasis, we propose that *lipl-4* represents such a gene, as elevated levels of *lipl-4*, although beneficial to the organism later in life, reduce reproductive success. The actions of LIPL-4 resulted in a trade-off between somatic maintenance and reproduction (Kirkwood, 2005). However, LIPL-4 function activates several different pathways that differ in terms of their effects on somatic proteostasis and reproduction. For example, *lipl-4(oe)* results in elevated levels of OEA that, in turn, activated NHR-49 and NHR-80. OEA did not affect proteostasis. However, NHR-49 and NHR-80 inversely regulated *lipl-*4 and vitellogenic gene expression (Seah et al., 2016; Figure 4), suggesting that OEA could regulate lipid resource allocation from yolk-bound lipoprotein to intestinal lipid droplets. In contrast, elevated levels of AA did not affect lipid distribution (Figure 6B) but rather were shown to induce autophagy in *C. elegans* and mammalian cells and extend *C. elegans* lifespan (O’Rourke et al., 2013). Moreover, *lipl-4(oe)* also resulted in a significant increase in DGLA (Folick et al., 2015). While DGLA, like AA, can induce autophagy and increase lifespan (O’Rourke et al., 2013), diet supplementation of DGLA resulted in a complete loss of germ cells and sterility, mediated by DGLA-derived epoxides (Watts and Browse, 2006; Deline et al., 2015). Finally, EPA did not affect proteostasis, autophagy, lifespan or reproduction in *C. elegans* (Watts and Browse, 2006; O’Rourke et al., 2013). Thus, the pleiotropic effects of LIPL-4 on reproduction and somatic maintenance are due to activation of different downstream pathways. Targeting metabolites that affect proteostasis but that have no or only mild effects on reproduction could artificially uncouple the two processes.

### AA-Modulated Proteostasis Collapse

How does LIPL-4 impact proteostasis? Diet supplementation of AA did not affect lipid distribution (Figure 6B) but was sufficient to recapitulate the impact of *lipl-4(oe)* on proteostasis (Figure 5 and Supplementary Figure S6B). Thus, although redistribution of lipids could activate several transcriptional programs that affect stress response pathways (Steinbaugh et al., 2015), it is less likely that lipid redistribution impacts somatic proteostasis. Accumulation of polyunsaturated fatty acids, such AA and DGLA, induce autophagy. Such induction itself could enhance proteostasis, for example, by removal of protein aggregates (Vilchez et al., 2014). However, AA could potentially activate other somatic maintenance pathways. For instance, exposure of HeLa cells to AA was shown to activate HSF1 and induce the expression of heat shock genes (Jurivich et al., 1994; Horikawa and Sakamoto, 2009; Balogh et al., 2013). Moreover, AA could be further metabolized to eicosanoids, including various prostaglandins that could also induce the heat shock response and affect the expression of heat shock genes in human cells (Amici et al., 1992; Balogh et al., 2013). Thus, AA supplementation has the potential to modulate proteostasis, a function that could also be conserved in humans. Determining when AA is required for proteostasis remodeling might help us elucidate the function this molecule serves.

Regulation of the proteostatic switch has a strong impact on the maintenance of somatic tissues (Shai et al., 2014). Given that this switch can impact the onset of protein aggregation, our findings could have consequences for the treatment of neurodegenerative diseases. The question, however, remains as to whether this proteostatic switch is conserved. While it is clear that proteostasis is limited in adults across species, currently there are only indications that the proteostatic switch itself is conserved (Shai et al., 2014; Labbadia and Morimoto, 2015b). These include the impact of the reproductive system on aging in flies and mice and the conservation of many of the players in these signaling pathways, including LIPL-4 (Flatt et al., 2008; Mason et al., 2009; Shai et al., 2014; Folick et al., 2015; Labbadia and Morimoto, 2015b). By finding ways to uncouple proteostatic collapse from the reproductive system, we might be able to target multiple age-dependent protein misfolding diseases with different etiologies but with similar underlying biology.

## Author Contributions

NShemesh and AB-Z designed the experiments and wrote the manuscript. NShemesh, NShai, LM and RK performed the experiments, analyzed the data and interpreted the results. NShemesh, NShai, LM, RK and AB-Z revised the text.

## Funding

This research was supported by a grant from the Legacy Heritage Biomedical Science Partnership Program of the Israel Science Foundation (grant No. 804/13; https://www.isf.org.il/#/) and by a grant from the Binational Science Foundation (grant No. 2009187; http://www.bsf.org.il). NShemesh was supported by Kreitman Negev scholarship and a Kreitman short-term post-doctoral scholarship.

## Conflict of Interest Statement

The authors declare that the research was conducted in the absence of any commercial or financial relationships that could be construed as a potential conflict of interest.
