# Supplementary material for: Uncoupling the Trade-Off between Somatic Proteostasis and Reproduction in Caenorhabditis elegans Models of Polyglutamine Diseases
Source: Front Mol Neurosci. 2017 Apr 20;10:101. doi: 10.3389/fnmol.2017.00101 (PMC5409330; doi:10.3389/fnmol.2017.00101)
Supplement: Supplementary file 1 [file Presentation_1.pdf]

Supplementary Material

Uncoupling the trade-off between somatic proteostasis and reproduction in *Caenorhabditis elegans* models of polyglutamine diseases

Netta Shemesh[1], Nadav Shai[1], Lana Meshnik[1], Rotem Katalan[1], Anat Ben-Zvi[1*]

*** Correspondence:** Anat Ben-Zvi: anatbz@bgu.ac.il

1 Supplementary Figures

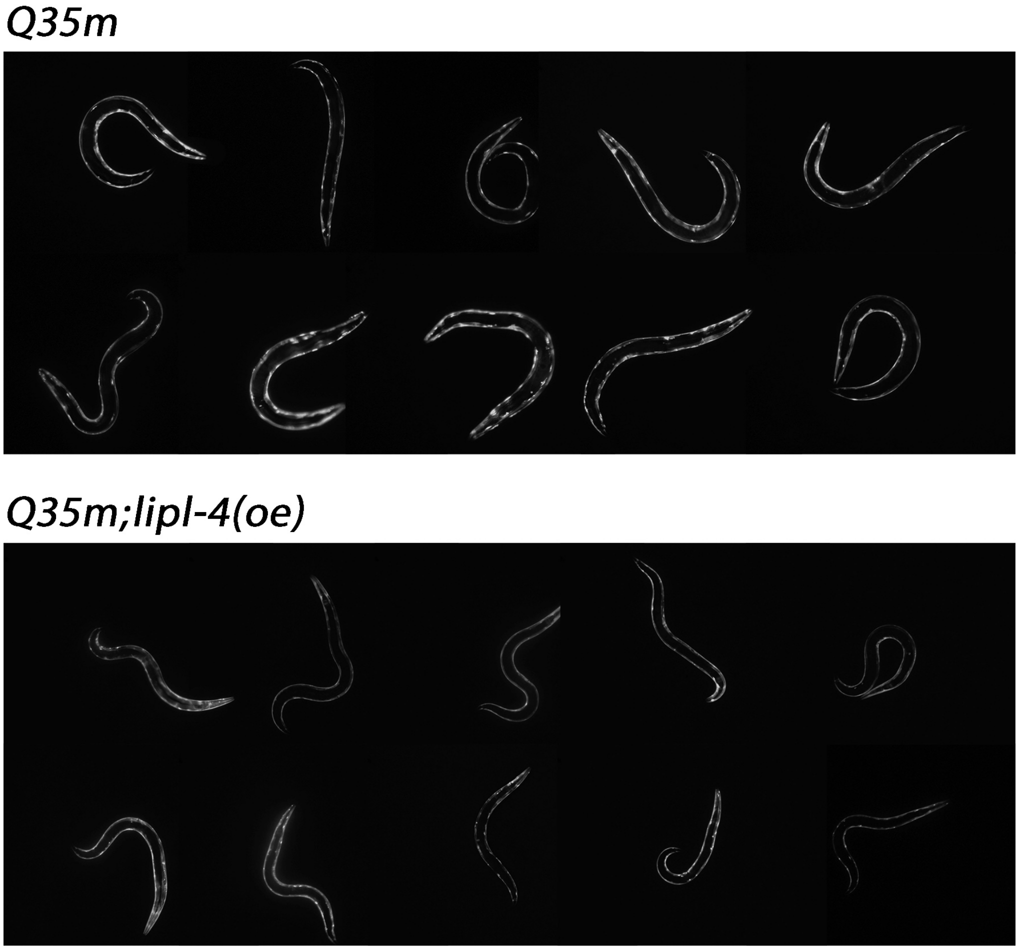

Supplementary Figure 1. Images of age-synchronized *Q35m;lipl-4(oe)* animals and their siblings on day 2 of adulthood.

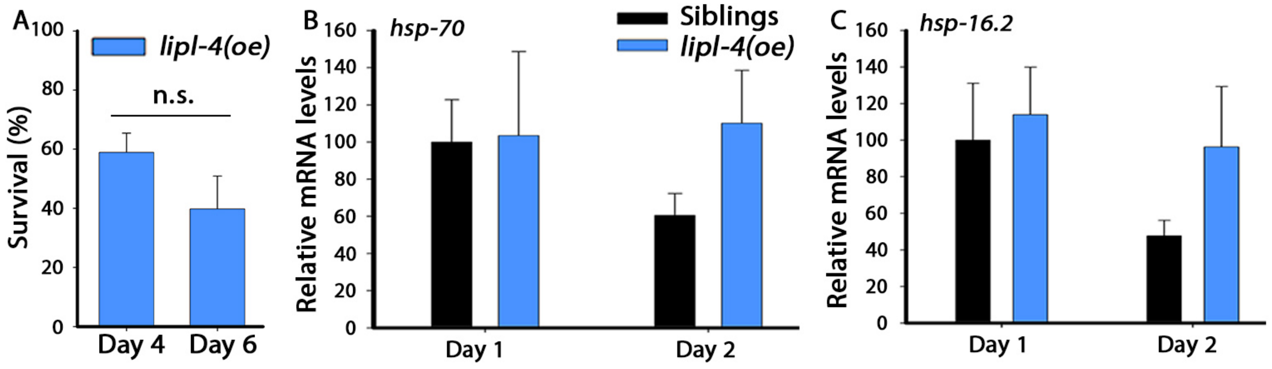

Supplementary Figure 2. Heat shock survival and activation was maintained in adult animals over-expressing *lipl-4*. (**A**) Thermo-resistance was examined in age-synchronized *lipl-4(oe)* and their siblings. Animals were subjected to heat shock (6 h at 37°C) at the indicated times and survival was assayed. Data was compared to *lipl-4(oe)* animals on day 2 of adulthood. (**B-C**) Quantification of *hsp-70* (B) and *hsp-16.2* (C) mRNA levels from age-synchronized *lipl-4*(*oe)* animals and their siblings following heat shock (90 min at 37°C). The data presented are normalized to treated siblings on day 1 of adulthood. (n.s.) no statistical significance.

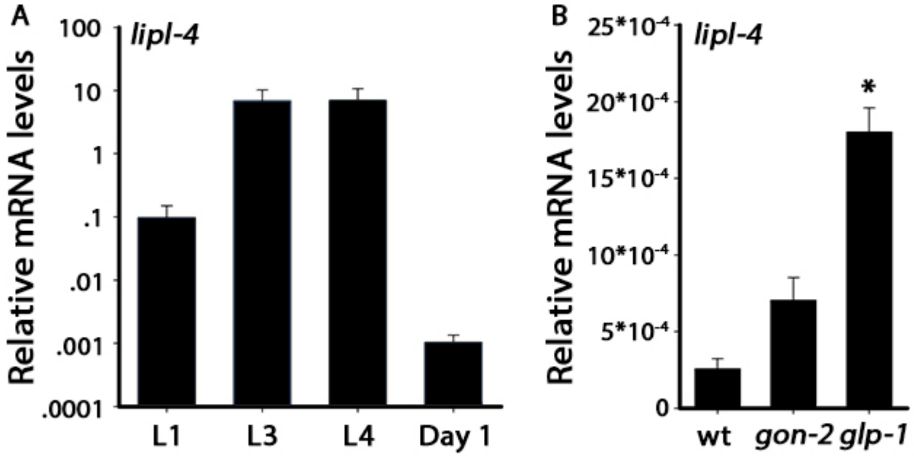

Supplementary Figure 3. *lipl-4* mRNA levels decline upon transition to adulthood. (**A**) Quantification of *lipl-4* mRNA levels from age-synchronized wild type animals at the indicated stages. (**B**) Relative mRNA levels of *lipl-4* from age-synchronized wild type (wt), *glp-1* or *gon-2* mutant animals on day 2 of adulthood. Data was compared to wt animals. (*) denotes $P<0.05$.

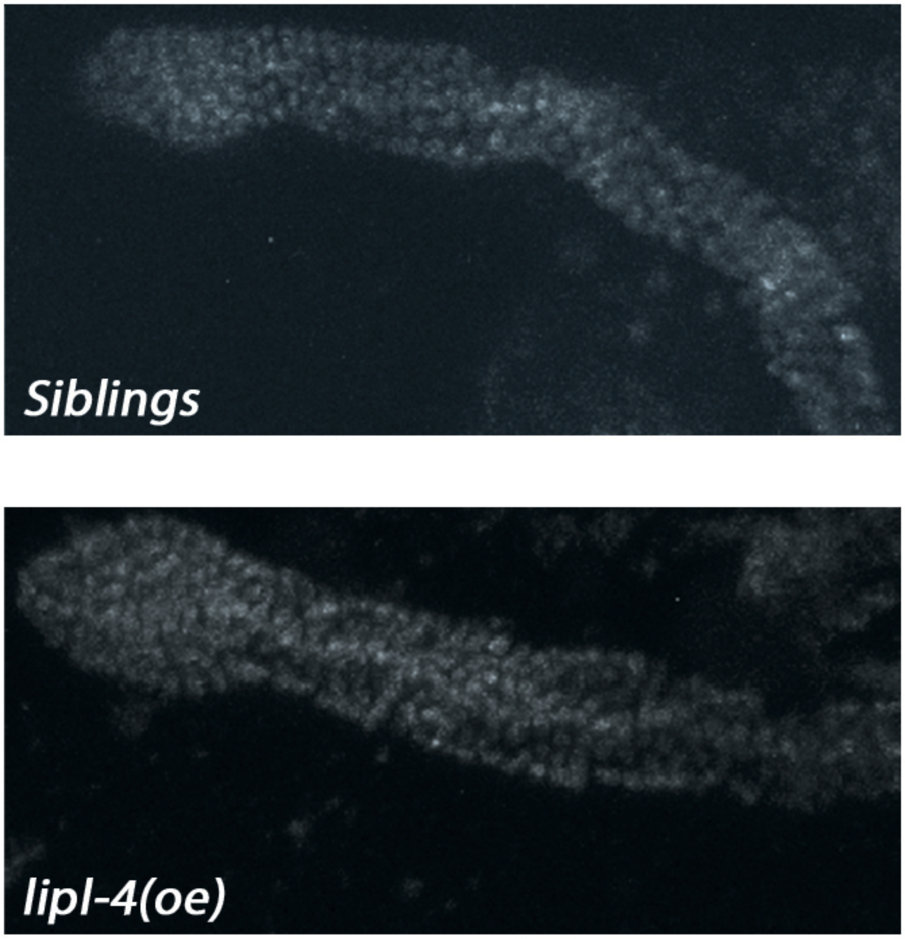

Supplementary Figure 4. ***lipl-4(oe)* did not induce germline proliferation arrest.** Representative images of germline cells from age-synchronized *lipl-4(oe)* animals and their siblings stained with DAPI on day 2 of adulthood.

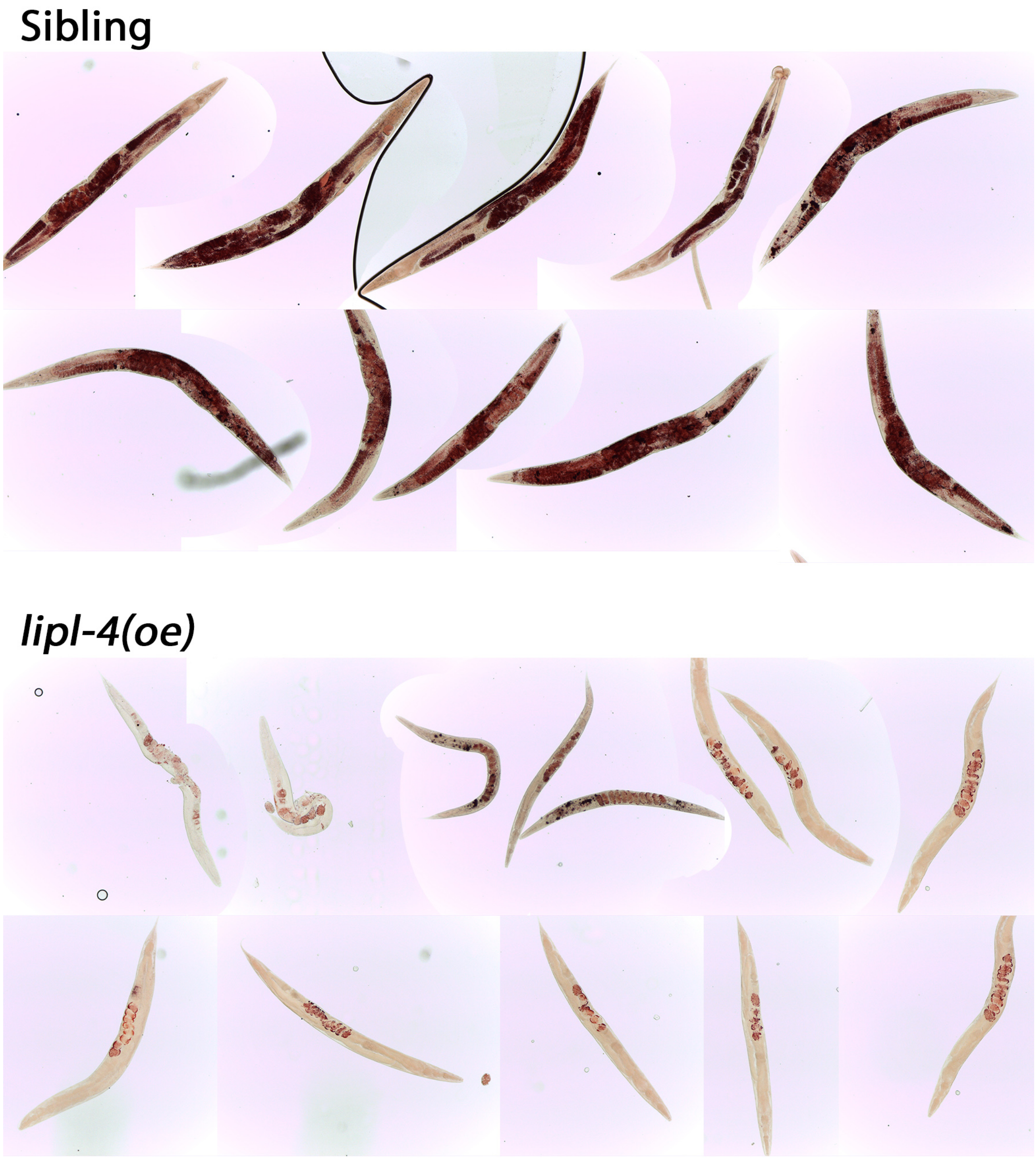

Supplementary Figure 5. *lipl-4(oe)* modulated fat stores. Age-synchronized *lipl-4(oe)* animals and their siblings were collected on day 2 of adulthood and total fat stores were analyzed using Oil-Red-O staining.

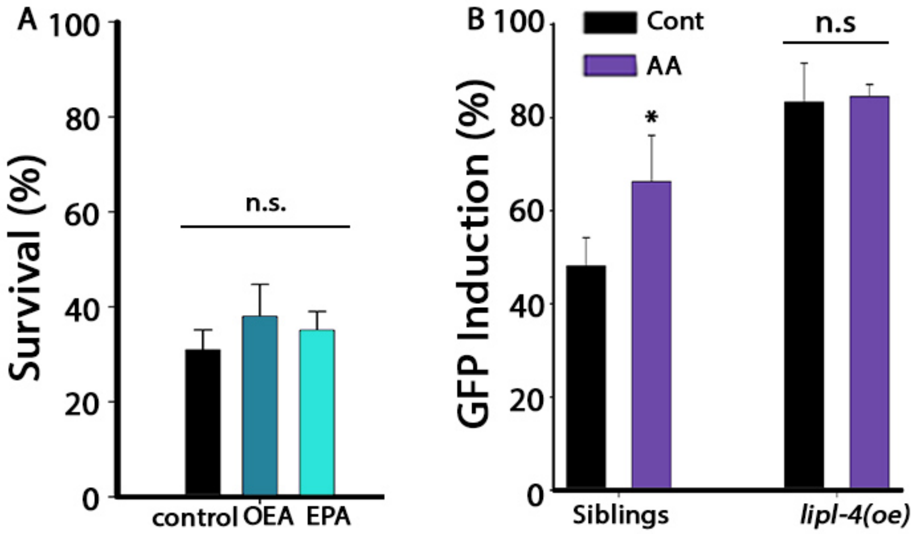

Supplementary Figure 6. Diet supplementation of AA but not OEA or EPA modulates proteostasis. (**A**) Thermo-resistance was examined in age-synchronized wild type animals grown on control (NP40), OEA- or EPA-supplemented NGM plates. Animals were subjected to heat shock (6 h at 37°C) and survival was assayed on day 2 of adulthood. Data was compared to NP40-supplemented plates (control). **(B)** Heat shock gene induction was examined in age-synchronized *lipl-4(oe)* animals and their siblings expressing *phsp-16.2::GFP.* Animals were grown on control (NP40) or AA-supplemented NGM plates, subjected to heat shock (90 min at 37°C) and scored for GFP expression. Data was compared to control plates (NP40). (n.s.) no statistical significance, (*) denotes $P<0.05$.

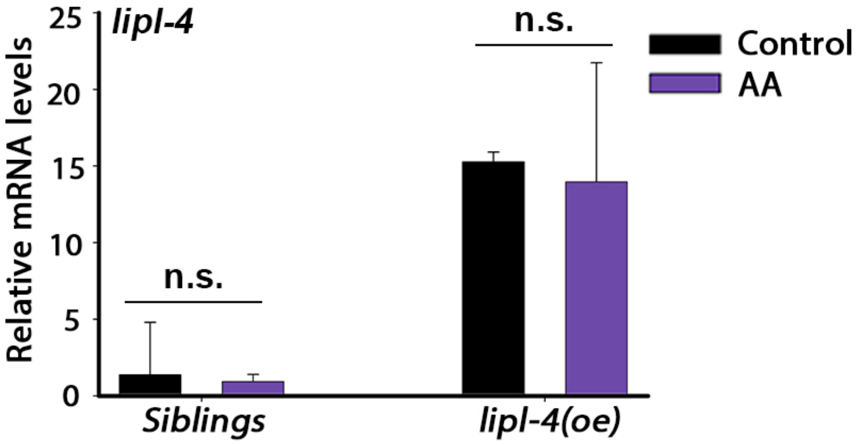

Supplementary Figure 7. AA did not induce *lipl-4* expression. Relative mRNA levels of *lipl-4* from age-synchronized *lipl-4(oe)* animals and their siblings grown on control (NP40) or AA-supplemented NGM plates. Data was compared to NP40-supplemented plates (control). (n.s.) no statistical significance.

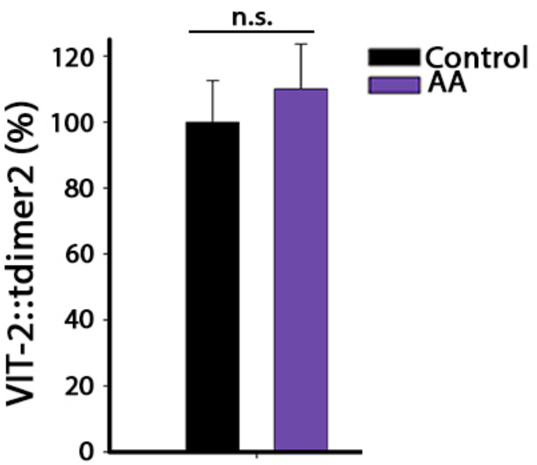

Supplementary Figure 8. AA did not modulate VIT-2 mobilization. Age-synchronized wild type animals expressing VIT-2::GFP were grown on control (NP40) or AA-supplemented NGM plates and imaged on the second day of adulthood. VIT-2::GFP levels were quantified from images using the ImageJ software and normalized to control. Data was compared to NP40-supplemented plates (control). (n.s.) no statistical significance.
